# Mortality in children with fulminant myocarditis: a six-year multicenter retrospective study

**DOI:** 10.1016/j.aicoj.2026.100030

**Published:** 2026-01-23

**Authors:** Lijun Yang, Wenting Zhao, Xuming Mo, Yucai Zhang, Jie Wang, Yanqin Cui, Zhenhua Liang, Yuxiong Guo, Wei Wang, Zhigang Liu, Daqing Ma, Ru Lin, Qiang Shu

**Affiliations:** a Department of Heart Failure and Mechanical Circulatory Support, Heart Institute, Children's Hospital, Zhejiang University School of Medicine, National Clinical Research Center for Child Health, Hangzhou, Zhejiang, China; b Department of Cardiac Surgery, Children's Hospital of Nanjing Medical University, Nanjing, Jiangsu, China; c Department of Pediatric Intensive Care Unit, Children's Hospital of Shanghai, Shanghai, China; d Department of Cardiac Intensive Care Unit, Children's Hospital of Henan Province, Zhengzhou, Henan, China; e Department of Cardiac Intensive Care Unit, Guangzhou Women and Children’s Hospital, Guangzhou, Guangdong, China; f Department of Pediatric Intensive Care Unit, The People’s Hospital of Guangxi Zhuang Autonomous Region, Nanning, Guangxi, China; g Department of Pediatric Intensive Care Unit, Guangdong Provincial People’s Hospital, Guangzhou, Guangdong, China; h Department of Extracorporeal Circulation, Shanghai Children’s Medical Center, Shanghai, China; i Perioperative and Systems Medicine Laboratory, Children’s Hospital, Zhejiang University School of Medicine, National Clinical Research Center for Child Health, Hangzhou, Zhejiang, China; j Department of Metabolism, Digestion and Reproduction, Faculty of Medicine, Imperial College London, London, United Kingdom; k Zhejiang Key Laboratory of Neonatal Diseases, Hangzhou, Zhejiang, China; l Division of Anesthetics, Pain Medicine & Intensive Care, Department of Surgery and Cancer, Faculty of Medicine, Imperial College London, Chelsea and Westminster Hospital, London, United Kingdom; m Department of Cardiac Surgery, Heart Institute, Children's Hospital, Zhejiang University School of Medicine, National Clinical Research Center for Child Health, Hangzhou, Zhejiang, China

**Keywords:** Pediatric fulminant myocarditis, Extracorporeal membrane oxygenation, Mortality, Risk factors, CK-MB, Lactate

## Abstract

**Background:**

Fulminant myocarditis (FM) in children can progress rapidly to cardiogenic shock, with high risk of mortality. Early recognition of prognostic markers is critical to guide timely escalation of circulatory support. This multicenter study sought to characterize clinical features and identify early predictors of in-hospital mortality in pediatric FM.

**Methods:**

We conducted a retrospective cohort study of patients <18 years with FM admitted to eight ECMO-capable pediatric intensive care units between January 2018 and August 2023. Clinical, biochemical, electrocardiographic, and echocardiographic variables were analyzed. Logistic regression was used to identify predictors of mortality, and receiver operating characteristic (ROC) curves were generated to assess discriminatory performance.

**Results:**

A total of 187 children were included; 157 (84.0%) required ECMO. In-hospital mortality was 16.6% (31/187). Univariate analysis identified elevated CK-MB, higher peak lactate, and ventricular tachycardia as associated with mortality. In multivariate analysis, peak lactate (AUC 0.791) and CK-MB (AUC 0.774) remained independent predictors. A combined model of peak lactate and ventricular tachycardia demonstrated moderate discrimination (AUC 0.772), whereas a composite model incorporating CK-MB, peak lactate, and ventricular tachycardia achieved the best predictive performance (AUC 0.815). Elevated lactate measured 12 h after initiation of extracorporeal membrane oxygenation or intensive conventional therapy further increased mortality risk (OR 1.219, 95% CI 1.004–1.481).

**Conclusion:**

Peak lactate, CK-MB, and ventricular tachycardia are early independent predictors of in-hospital mortality in pediatric FM. Persistent hyperlactatemia within 12 h of advanced support provides additional prognostic value and may assist clinicians in early risk stratification.

## Introduction

Pediatric fulminant myocarditis (FM) is a rapidly progressing form of acute myocarditis that often results in life-threatening conditions such as cardiogenic shock, ventricular arrhythmias, multiorgan failure, and sudden death [1]. The condition demands urgent intervention, often requiring inotropic drugs, vasoactive agents, or mechanical circulatory support [1]. While the incidence of pediatric FM is relatively low, its associated mortality is high, contributing to approximately 2% of all pediatric deaths [2,3]. This elevated mortality rate is primarily attributed to the rapid progression of the disease, typically manifested as circulatory collapse, which markedly shortens the window for establishing a diagnosis and initiating effective therapy [4]. This delayed identification and intervention often result in misdiagnosis or inadequate treatment during the critical early phase of the illness, which worsens patient outcomes.

Despite its high initial mortality, pediatric FM has a relatively favorable long-term prognosis once the acute high-risk period is managed effectively [5]. If proper supportive care, such as mechanical circulatory support, is administered in time, many patients can recover and achieve normal or near-normal quality of life. The advent and widespread application of extracorporeal membrane oxygenation (ECMO) have significantly improved survival rates in patients with severe forms of FM [6]. ECMO serves as an essential tool in supporting the heart and lungs during the most critical phases of the disease, particularly when conventional therapies are insufficient.

Few studies have comprehensively explored the factors that contribute to mortality, especially those that can be detected early in the disease process. The aim of this multicenter retrospective study, conducted across eight high-volume ECMO centers in China, is to identify key clinical and laboratory markers associated with in-hospital mortality in pediatric patients with FM. By understanding these risk factors, we hope to identify early predictors of poor outcomes and guide timely interventions, thereby reducing mortality and improving patient outcomes in this high-risk group.

## Methods

### Study population

The cohort enrolled 187 children diagnosed with pediatric FM, as identified from the electronic medical records of the participating centers. FM was diagnosed clinically, as an endomyocardial biopsy was not performed. The diagnostic criteria for pediatric FM were as follows: (1) acute onset and rapid progression, often preceded within days to 2–4 weeks by a prodromal infection, autoimmune flare, new medication exposure, or toxin exposure; (2) presentation with critical illness characterized by abrupt cardiogenic shock, acute heart failure, or aborted sudden cardiac death requiring inotropic support and/or mechanical circulatory support, frequently accompanied by multi-organ dysfunction; (3) marked elevation of cardiac biomarkers, including cardiac troponin I/T, high-sensitivity troponin I/T, and creatine kinase-MB (CK-MB); (4) electrocardiographic abnormalities such as arrhythmias, low-voltage QRS, or ST-segment/T-wave changes; (5) echocardiographic evidence of myocardial edema, diffuse or segmental hypokinesia, and reduced left-ventricular systolic function.

Inclusion criteria were: (1) patients aged <18 years; (2) diagnosis of FM meeting the above criteria, defined as acute myocarditis with rapid progression to cardiogenic shock requiring inotropic or vasoactive support and/or mechanical circulatory support (ECMO was the only modality used in this cohort); and (3) cases with ventricular arrhythmias or cardiac arrest requiring cardiopulmonary resuscitation (CPR). Exclusion criteria were: (1) neonates; (2) patients with myocardial infarction, congenital heart disease, or other primary cardiac diseases that could confound the analysis.

This study was approved by the Ethics Committee of the Children’s Hospital of Zhejiang University School of Medicine, Hangzhou, China (approval number 2020-IRB-006). Given the retrospective design and use of anonymized data, the requirement for informed consent was waived by the ethics committees of all participating centers. We conducted a multicenter, retrospective cohort study involving data from eight tertiary medical centers in China, each of which is equipped with advanced capabilities for pediatric ECMO support. The study period (January 2018–August 2023) was chosen to reflect the contemporary era of pediatric ECMO management across the participating centers. Since 2020, the eight centers jointly established a multicenter framework and unified inclusion criteria for fulminant myocarditis. Earlier electronic health records (prior to 2018) were largely incomplete for key biochemical and hemodynamic indicators such as lactate and SVO₂ at 12 and 24 h. Therefore, data collection was restricted to cases from 2018 onward, with August 2023 serving as the prespecified data cutoff to ensure consistency and completeness across centers.

### ECMO and intensive conventional therapy (ICT) indications

Cardiac index (CI) was primarily measured using a Swan–Ganz (pulmonary artery) catheter. When invasive monitoring was not feasible, CI was estimated by transthoracic echocardiography using the LVOT diameter and velocity–time integral (VTI) to calculate stroke volume, multiplied by heart rate and normalized to body surface area. In some older children, a percutaneous cardiac function monitoring device was used as a supplementary reference. The decision to initiate ECMO was based on the following clinical criteria: (1) CI < 2.0 L/min/m^2^; (2) metabolic acidosis, as indicated by a base excess ≤−5 mmol/L and a lactate level >3.0 mmol/L; (3) signs of end-organ failure, including urinary output <0.5 mL/kg/h; or (4) mean arterial pressure thresholds for age (infants <50 mmHg, children <60 mmHg) when these conditions persist for more than 3 h despite optimized inotropic therapy. Additionally, ECMO was indicated for patients suffering cardiac arrest and necessitating cardiopulmonary resuscitation.

All pediatric FM patients were categorized into ECMO and non-ECMO groups. Intensive conventional therapy (ICT) refers to the use of multiple vasoactive or inotropic drugs and/or temporary pacemaker implantation as supportive management before ECMO initiation. The initiation of ECMO or ICT was defined as the time point for assessing treatment efficacy. All ECMO procedures were performed using peripheral cannulation. Among the 157 patients supported with ECMO, 58 underwent percutaneous cannulation and 99 underwent surgical cut-down.

### Data collection

Clinical and demographic data were extracted from the medical records of all enrolled patients. Demographic variables included age, sex, and weight. Clinical characteristics included initial symptoms, Glasgow Coma Scale (GCS) score, CPR and extracorporeal CPR (ECPR) status, use of continuous renal replacement therapy (CRRT), and the duration of mechanical ventilation, ICU stay, and overall hospital stay. Vasoactive agents, including adrenaline, dopamine, dobutamine, milrinone, noradrenaline, as well as vasoactive–inotropic score (VIS) [7] were collected.

Laboratory values measured within the first 24 h of ICU admission were recorded, including blood lactate levels, platelet count, prothrombin time (PT), international normalized ratio (INR), alanine aminotransferase (ALT), aspartate aminotransferase (AST), bilirubin, creatinine, and cardiac biomarkers, such as cardiac troponins (cTnI, cTnT), CK-MB, brain natriuretic peptide (BNP), and N-terminal pro-brain natriuretic peptide (pro-BNP).

Additionally, electrocardiographic (ECG) abnormalities, chest X-ray findings, and echocardiographic data, including left ventricular ejection fraction (LVEF), were reviewed. To assess treatment efficacy, parameters such as central venous oxygen saturation (SVO_2_), urine output, and lactate levels at 12 and 24 h following ECMO or ICT initiation were also recorded.

### Mortality and complication definitions

In-hospital mortality was defined as death occurring during the index hospitalization. Major complications were those with substantial clinical impact, including neurological injury (intracranial hemorrhage or cerebral infarction), acute liver failure⁷, acute kidney injury (RIFLE stage III or above, or requiring dialysis), and severe limb complications such as ischemia leading to necrosis or amputation. Pulmonary edema was defined as bilateral pulmonary infiltrates without evidence of infection, confirmed radiographically.

Continuous renal-replacement therapy (CRRT) was initiated in patients with acute kidney injury presenting with oliguria or anuria (<0.5 mL/kg/h) when accompanied by one or more of the following conditions: fluid overload >10%, severe electrolyte or metabolic derangements refractory to medical therapy (e.g., hyperammonemia), persistent severe metabolic acidosis, uremic complications (e.g., encephalopathy, pericardial effusion, pulmonary edema), intoxication with dialyzable agents, septic shock requiring removal of inflammatory mediators, or the need to secure intravascular volume capacity for essential drug therapy or nutrition.

### Statistical analysis

Continuous variables were analyzed for normality using the Shapiro-Wilk test. Data were presented as median with interquartile range (IQR). Differences between groups were assessed using the Student’s t-test for normally distributed data and the Mann-Whitney U test for skewed data. Categorical variables were expressed as numbers with percentages and compared using Fisher's exact test, with Bonferroni correction for multiple comparisons when necessary.

Univariate analysis was first performed to identify significant variables associated with in-hospital mortality. Variables for multivariate logistic regression were selected based on both clinical relevance and statistical significance in univariate analyses. Age, weight, sex, ECPR, peak lactate, PLT, PT, VIS, AST, ALT, creatinine, LVEF, and ventricular tachycardia and CK-MB were investigated in regression models. CK-MB was retained as a myocarditis-specific biomarker reflecting the extent of myocardial injury in the fulminant subtype. The results of the multivariate regression are presented as odds ratios (OR) with 95% confidence intervals (CI). To evaluate the predictive accuracy of the identified risk factors, receiver operating characteristic (ROC) curves were generated, and the area under the curve (AUC) was calculated. The optimal cut-off was determined as the value that maximized the Youden index (sensitivity + specificity − 1) on the ROC curve.

A p-value of <0.05 was considered statistically significant. All statistical analyses were performed using SPSS version 21.0 (IBM, Armonk, NY, USA).

## Results

### Demographics and clinical characteristics

**Fig. 1:**
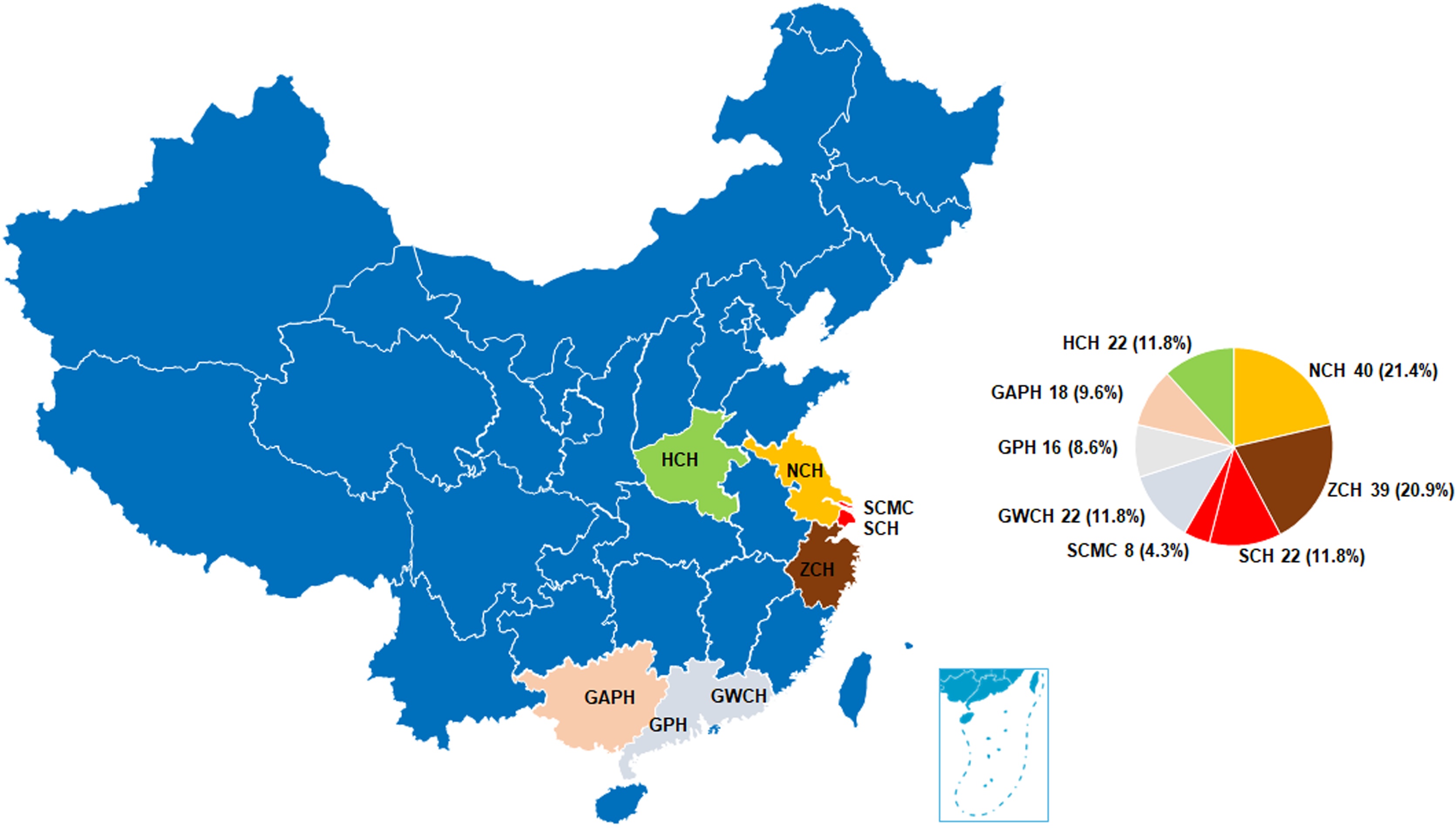
Temporal distribution of pediatric fulminant myocarditis cases across eight tertiary ECMO centers in China (January 2018–August 2023; n = 187). Each colored line corresponds to one participating center: Zhejiang University Children’s Hospital (ZJU), Nanjing Medical University Children’s Hospital (NJMU), Shanghai Children’s Hospital (SH), Children’s Hospital of Henan Province (HEN), Guangzhou Women and Children’s Hospital (GWH), The People’s Hospital of Guangxi Zhuang Autonomous Region (GX), Guangdong Provincial People’s Hospital (GP), and Shanghai Children’s Medical Center (SCMC).

A total of 187 pediatric patients with fulminant myocarditis (FM) were included in this study, conducted across eight high-volume ECMO centers in China, spanning from January 2018 to August 2023 (Fig. 1). The cohort had a median age of 7.8 years (IQR: 5.0–10.4 years), with a median weight of 24 kg (IQR: 18–35 kg) and with 55.5% of the cohort being female (Table1) Among the patients, 38.5% (72/187) experienced cardiac arrest, and 21.9% (41/187) received ECMO during cardiopulmonary resuscitation (ECPR). A total of 157 patients (84.0%) received ECMO support during the course of their treatment, and 19.8% (37/187) of patients required pacemaker implantation. Continuous renal replacement therapy (CRRT) was utilized in 24.6% of cases (46/187). Vasoactive-inotropic data were available for 156 patients. Epinephrine was administered in 105/156 (67.3%), making it the primary vasoactive agent. Dopamine accounted for 24.4 %, whereas norepinephrine, milrinone, and dobutamine were employed infrequently (19.2%, 19.2%, and 24.4%, respectively).

**Table 1:** Demographic data, characteristics of children with FM. Categorical variables are depicted as N (%), and continuous variables as median (IQR).

Variables	Overall (187)	Survival (156)	Death (31)	p
Age(year)	7.8 (5, 10.4)	8.2 (5.1, 10.5)	6.1 (4, 9.8)	0.058
Male	85/187 (45.5%)	69/156 (44.2%)	16/31 (48.4%)	0.451
Weight (kg)	24 (18, 35)	25 (18, 37)	21 (16, 30)	0.108
CPR (%)	72/187 (38.5%)	49/156 (31.4%)	23/31 (74.2%)	<0.001
ECPR (%)	41/187 (21.9%)	30/156 (19.2%)	11/31 (35.5%)	0.046
ECMO (%)	157/187 (84.0%)	129/156 (82.7%)	28/31 (90.3%)	0.290
Pacemaker (%)	37/187 (19.8%)	28/156 (17.9%)	9/31 (29.0%)	0.253
CRRT (%)	46/187 (24.6%)	31/156 (19.9%)	15/31 (48.4%)	0.003
Onset symptoms				
Respiratory	44/187 (23.5%)	35/156 (22.4%)	9/31 (29.0%)	0.514
Circulatory	77/187 (41.2%)	64/156 (41.0%)	13/31 (41.9%)	0.600
Digestive	96/187 (51.3%)	80/156 (51.3%)	16/31 (51.6%)	0.663
Neurological	52/187 (27.8%)	42/156 (26.9%)	10/31 (32.3%)	0.481
Others	13/187 (7.0%)	10/156 (6.4%)	3/31 (9.7%)	0.499

ECMO, extracorporeal membrane oxygenation; CPR, cardiopulmonary resuscitation; ECPR, ECMO, cardiopulmonary resuscitation; CRRT, continuous renal replacement therapy.

The most common initial symptom was gastrointestinal in nature, observed in 51.3% (96/187) of patients, followed by cardiovascular (41.2%), neurological (27.8%), and respiratory symptoms (23.5%). The predominant symptoms included fever (71 cases), vomiting (54 cases), abdominal pain (30 cases), chest tightness (28 cases), convulsions (25 cases), fatigue (20 cases), chest pain (14 cases), dizziness (11 cases), and shortness of breath (11 cases).

### Mortality and ICU outcomes

The overall in-hospital mortality rate in this cohort was 16.6% (31/187). The median duration of mechanical ventilation was 7.0 days (IQR: 4.0–11.0 days), and the median length of ICU stay was 12.0 days (IQR: 7.0–18.0 days) (Table 2). The median total length of hospital stay was 24.0 days (IQR: 13.5–32.0 days). Among the 31 patients who died during hospitalization, 28 were from the ECMO group (17.8% of the ECMO cohort) and 3 from the non-ECMO group (10% of the non-ECMO cohort). Of the 31 children who died, 11 were transferred on ECMO after diagnoses elsewhere and succumbed to irreversible multi-organ failure or brain death; 7 developed massive intracranial hemorrhage/infarction during ECMO; 4 had irreversible cardiac failure failed to wean ECMO; 3 were misdirected to other departments with resultant treatment delay; 3 progressed to multi-organ failure while receiving conventional therapy without ECMO; and 3 had care withdrawn because of financial constraints.

**Table 2:** Outcomes of children with FM. Categorical variables are depicted as N (%), and continuous variables as median (IQR).

Variables	Overall (187)	Survival (156)	Death (31)	p
Length of ventilation(d)	7.0 (4.0, 11.0)	7.0 (5.0, 10.8)	5.0 (1.0, 16.0)	0.499
Length of ICU stay(d)	12.0 (7.0, 18.0)	12.0 (8.0, 18.0)	8.0 (2.0, 18.0)	0.022
Length of hospital stay(d)	24.0 (13.5, 32.0)	25.0 (17.0, 34.0)	8.0 (2.0, 18.0)	<0.001
Major complications				
Severe neurological complications	25/187 (13.4%)	6/156 (3.8%)	19/31 (61.3%)	<0.001
Hepatic failure	16/187 (8.6%)	5/156 (3.2%)	11/31 (35.5%)	<0.001
Renal failure	25/187 (13.4%)	10/156 (6.4%)	15/31 (48.4%)	<0.001
Severe limb complications	7/187 (3.7%)	3/156 (1.9%)	4/31 (12.9%)	0.015

ICU, intensive care unit.

A detailed analysis of complications revealed that renal failure (13.4%, 25/187) and severe neurological complications (13.4%, 25/187) were the most common major complications. Other significant complications included hepatic failure (8.6%, 16/187) and extremity injuries (3.7%, 7/187). Renal failure and cerebral complications were more frequently observed in the death group compared to the survival group (p = 0.001 and p = 0.001, respectively). In contrast, the survival group exhibited a notably lower incidence of major complications.

### Electrocardiographic and echocardiographic findings

Electrocardiographic (ECG) abnormalities were present in 92.2% (154/167) of patients, with atrioventricular block (AVB) observed in 56.3%, ST-segment depression in 52.7%, sinus tachycardia in 47.9%, and ventricular tachycardia (VT) in 18.6% (Table 3). The most common form of AVB was complete AV block, which accounted for nearly one-third of AVB cases. Chest X-ray revealed cardiac enlargement in 28.1% (48/171) of patients, while 33.9% (58/171) exhibited pulmonary edema, suggesting secondary pulmonary hypertension related to acute left ventricular dysfunction. Interestingly, 36.3% (36/99) of patients were initially misdiagnosed with pneumonia, which delayed appropriate treatment.

**Table 3:** Variables related to mortality. Categorical variables are depicted as N (%), and continuous variables as median (IQR).

Variables (n^a^)	Overall	Survival	Death	p
Vital signs				
MAP (170)	59 (50, 65)	61 (50, 65)	56 (39, 65)	0.130
Systolic BP (170)	76 (66, 86)	76 (68, 86)	68 (52, 85)	0.055
Diastolic BP (170)	50 (42, 57)	51 (43, 57)	46 (36, 56)	0.131
Fastest HR (162)	154 (130, 181)	150 (126, 175)	170 (152, 200)	0.001
Lowest HR (162)	65 (46, 94)	65 (49, 91)	73 (29, 107)	0.06
GCS (104)	15 (8, 15)	15 (11, 15)	8 (4, 13)	0.001
Vasoactive agents (μg/kg/min, n = 156)				
Adrenalin	0.1 (0.0, 0.2)	0.1 (0.0, 0.2)	0.1 (0.1, 0.6)	0.006
Noradrenalin	0.0 (0.0, 0.0)	0.0 (0.0, 0.0)	0.0 (0.0, 0.1)	0.040
Dopamine	0.0 (0.0, 5.5)	0.0 (0.0, 5.1)	0.0 (0.0, 7.4)	0.728
Dobutamine	0.0 (0.0, 0.0)	0.0 (0.0, 0.0)	0.0 (0.0, 5.0)	0.277
Milrinone	0.0 (0.0, 0.0)	0.0 (0.0, 0.0)	0.0 (0.0, 0.0)	0.564
VIS	19.2 (5.9, 30.0)	15.8 (5.1, 29.0)	24.5 (13.9, 60.0)	0.012
Lab tests (n, unit)				
Lactate peak (164, mmol/L)	7.0 (4.1, 13.2)	6.1 (3.8, 10.8)	13.2 (7.9, 15.3)	<0.001
BE (168, mmol/L)	−9.2 (−15.3, −4.8)	−8.7 (−13.4, −4.5)	−13.4 (−19.7, −8.5)	0.037
PH (169, mmol/L)	7.24 (7.13, 7.33)	7.27 (7.13, 7.34)	7.16 (6.91, 7.23)	0.572
PLT (178, *10^9^/L)	100 (62, 157)	106 (65, 165)	64 (18, 145)	0.929
PT (172, s)	19.5 (16.0, 28.9)	19.0 (16.0, 27.6)	22.4 (17.6, 44.3)	0.039
INR (167)	1.73 (1.37, 2.62)	1.73 (1.36, 2.50)	1.89 (1.54, 3.99)	0.104
ALT (179, U/L)	210 (69, 1079)	136 (61, 733)	837 (223, 1736)	0.002
AST (174, U/L)	308 (111, 1574)	239 (96, 1136)	1380 (275, 4202)	<0.001
Bilirubin (171, μmol/L)	20.0 (12.0, 37.4)	17.9 (12.0, 32.8)	23.5 (12.1, 60.8)	0.352
Creatine (179, μmol/L)	100 (58, 136)	63 (47, 93)	95 (62, 178)	0.146
TnT (54, ng/mL)	2.08 (1.03, 3.55)	2.07 (0.84, 3.60)	2.39 (1.62,3.74)	0.688
TnI (126, ng/mL)	9.03 (3.19, 13.90)	9.58 (3.29, 18.00)	13.49 (8.14,28.48)	0.147
CK-MB (131, U/L)	112 (59, 261)	105 (52, 212)	292 (123, 889)	<0.001
BNP (79, pg/mL)	3635 (1561, 5018)	3877 (1557, 5244)	3272 (1473, 9707)	0.771
Pro-BNP (52, pg/mL)	10998 (5753, 16889)	14342 (8031, 20312)	16552 (5431, 24516)	0.662
Electrocardiographic (n = 162)				
Abnormal ECG	154/167 (92.2%)	132/141 (93.6%)	22/26 (84.6%)	0.240
Sinus tachycardia	80/167 (47.9%)	64/139 (46.0%)	16/28 (57.1%)	0.283
Ventricular tachycardia	31/167 (18.6%)	22/139 (15.8%)	9/28 (32.1%)	0.043
Supraventricular tachycardia	6/167 (3.6%)	5/139 (3.6%)	1/28 (3.6%)	1
Ventricular premature beat	13/167 (7.8%)	12/139 (8.6%)	1/28 (3.6%)	0.536
Ventricular fibrillation	9/167 (5.4%)	6/139 (4.3%)	3/28 (10.7%)	0.363
III^。^AVB	32/167 (19.2%)	29/139 (20.9%)	3/28 (10.7%)	0.213
Other AVB	62/167 (37.1%)	55/139 (39.6%)	7/28 (25.0%)	0.145
Depression of ST segment	88/167 (52.7%)	80/139 (57.6%)	8/28 (28.6%)	0.005
Elevation of ST segment	13/167 (7.8%)	12/139 (8.6%)	1/28 (3.6%)	0.599
QT interval prolongation	9/167 (5.4%)	7/139 (5.0%)	2/28 (7.2%)	1
QRS low voltage	15/167 (9.0%)	12/139 (8.6%)	3/28 (10.7%)	1
Abnormal Q wave	24/167 (14.4%)	22/139 (15.8%)	2/28 (7.2%)	0.368
Chest X-ray (n = 171)				
Enlarged heart shadow	48/171 (28.1%)	41/142 (28.9%)	7/29 (24.1%)	0.605
Pulmonary edema	58/171 (33.9%)	46/142 (32.4%)	12/29 (41.4%)	0.352
Echocardiography (n = 130)				
LVEF (%)	31 (20, 43)	32 (22, 44)	24 (16, 38)	0.05

MAP, mean arterial pressure; BP, blood pressure; HR, heart rate; GCS, Glasgow coma scale; VIS, vasoactive inotropic score; BE, base excess; PLT, platelet count; PT, prothrombin time; INR, international normalized ratio; ALT, alanine aminotransferase; AST, aspartate aminotransferase; TnT, troponin-T; TnI, troponin-I; CK-MB, creatine kinase-MB; BNP, brain natriuretic peptide; ECG, electrocardiography; AVB, atrioventricular block. LVEF, left ventricular ejection fraction.

a ”n” represents the number of cases of each variable. Low utilization of certain agents (e.g., milrinone) resulted in median and IQR values of 0.0, reflecting that most patients did not receive these medications.

Echocardiographic data showed that the worst left ventricular ejection fraction (LVEF) within the first 24 h of ICU admission was 31% (20%, 43%), further highlighting the severe cardiac dysfunction in this cohort.

### Comparison between ECMO and non-ECMO groups

Of the 187 patients in the study, 157 (84%) were treated with ECMO, which was associated with more severe clinical conditions compared to the non-ECMO group. Key clinical parameters, including CK-MB, creatinine, peak lactate levels, PT, and LVEF, were significantly worse in the ECMO group, indicating that these patients had more profound hemodynamic instability. In the non-ECMO group, only 4 cases experienced lactate levels ≥3 mmol/L for more than 3 h. The median time from ICU admission to ECMO initiation was 4 h (IQR: 2–10 h), and the median duration of ECMO support was 128 h (approximately 5 days). In the ECMO group, 58 underwent percutaneous cannulation and 99 surgical cut-down; all cannulations were peripheral.

### SVO_2_, lactate, and urine output

Treatment efficacy parameters, such as central venous oxygen saturation (SVO_2_), lactate levels, and urine output, were closely linked to mortality. At 12 h after ECMO or ICT initiation, SVO_2_ levels <65%, lactate levels >5 mmol/L, and urine output <1 ml/kg/h were associated with significantly higher mortality rates. In particular, the 12-h lactate levels were found to be a particularly sensitive indicator of mortality. Specifically, 12-h lactate levels greater than 5 mmol/L were associated with a markedly higher mortality risk (p = 0.009) (Table 4).

**Table 4:** Early treatment response parameters at 12 and 24 h after initiation of ECMO or ICT. Categorical variables are depicted as N (%), and continuous variables as median (IQR).

	Overall	Survival	Death	p
12 h after ECMO/ICT				
SVO_2_ (106, %)	71 (61, 80)	73 (66, 80)	58 (54, 70)	0.001
SVO_2_ <65%	33/111 (29.7%)	21/95 (22.1%)	12/16 (75.0%)	<0.001
Lactate (147, mmol/L)	1.8 (1.1, 3.5)	1.6 (1.0, 3.2)	3.0 (1.9, 10.2)	0.001
Lactate >5 mmol/L	28/151 (18.5%)	19/129 (14.7%)	9/22 (40.9%)	0.009
Urine output (154, ml/kg/h)	2.2 (1.1,3.9)	2.3 (1.3,4.0)	1.1 (0.1,2.8)	0.022
Urine output <1 ml/kg/h	36/160 (22.5%)	23/135 (17.0%)	13/25 (52.0%)	<0.001
24 h after ECMO/ICT				
SVO_2_ (115, %)	71 (64,80)	71 (66,80)	65 (58,81)	0.129
SVO_2_ <65%	26/115 (22.6%)	19/101 (18.8%)	7/14 (50.0%)	0.023
Lactate (150, mmol/L)	1.6 (1.0,2.2)	1.5 (0.9,2.0)	2.6 (1.7,6.9)	<0.001
Lactate >5 mmol/L	19/150 (12.7%)	13/129 (10.1%)	6/21 (28.6%)	0.045
Urine output (149, ml/kg/h)	3.1 ± 2.6	3.2 ± 2.6	2.5 ± 2.6	0.291
Urine output <1 ml/kg/h	32/150 (21.3%)	25/130 (19.2%)	7/20 (35.0%)	0.190

SVO_2,_ central venous oxygen saturation.

At 24 h, SVO_2_, lactate, and urine output were still predictive of outcomes. SVO_2_ < 65% at 24 h was associated with higher mortality (p = 0.023), while the lactate levels remained a significant predictor of mortality (p = 0.045) (Table 4).

### Mortality risk factors

**Fig. 2:**
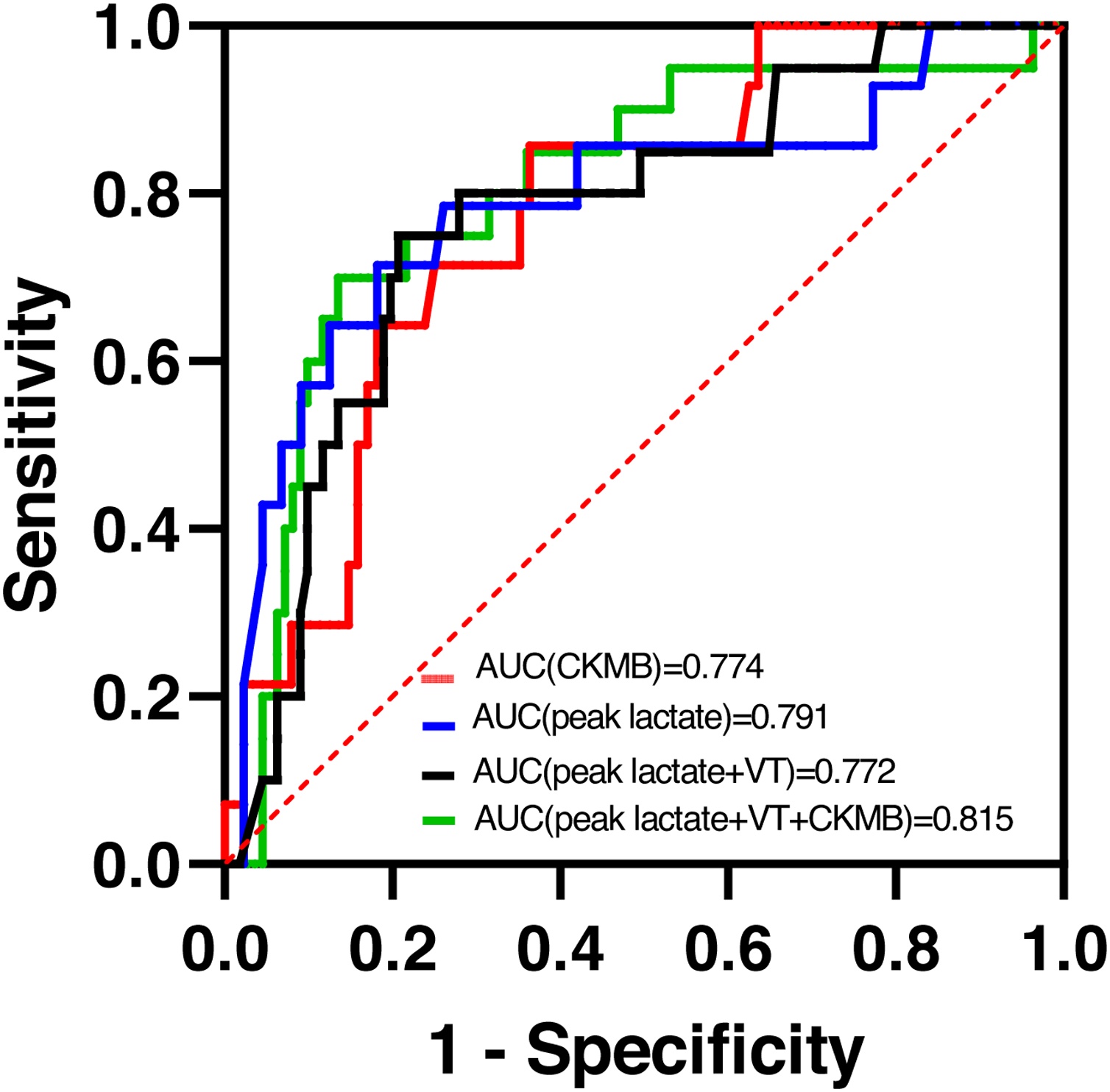
Receiver operating characteristic (ROC) curves for predicting in-hospital mortality in pediatric fulminant myocarditis. Data were shown as the ROC curves for peak lactate, ventricular tachycardia (VT) plus peak lactate, CK-MB alone, and the combined multivariable model incorporating peak lactate, VT, and CK-MB. Sensitivity is plotted against 1 – specificity. Inclusion of CK-MB modestly improves discrimination for identifying patients at high risk of mortality.

Univariate logistic regression was performed to screen clinical and laboratory variables potentially associated with in-hospital mortality (Table 3). Factors showing significant associations included elevated CK-MB (OR 1.003, 95% CI 1.001–1.006), peak lactate (OR 1.194, 95% CI 1.037–1.374), and the presence of ventricular tachycardia (OR 6.796, 95% CI 1.167–39.569). In multivariate analysis, peak lactate (AUC 0.791) and CK-MB (AUC 0.774) remained independent predictors of mortality. A combined model of peak lactate and ventricular tachycardia demonstrated moderate discriminatory ability (AUC 0.772), whereas a composite model incorporating CK-MB, peak lactate, and ventricular tachycardia achieved the highest predictive performance (AUC 0.815) (Fig. 2). Additionally, elevated lactate measured 12 h after initiation of ECMO or ICT was associated with increased mortality risk (OR 1.219, 95% CI 1.004–1.481), supporting its use as a dynamic indicator of treatment response (Table 4).

### Predictive models and ROC analysis

Receiver operating characteristic analysis demonstrated that the AUC for predicting mortality using peak lactate levels was 0.769, and the AUC for the combined prediction model (including peak lactate and ventricular tachycardia) was 0.772. When including CK-MB in the analysis, the AUC improved to 0.815, further supporting the clinical relevance of these early biomarkers in predicting mortality in pediatric FM (Fig. 2).

## Discussion

In this multicenter retrospective study, the in-hospital mortality of pediatric FM was relatively low (16.6%), likely reflecting the high utilization of ECMO support (84% of patients). We identified elevated CK-MB, high peak lactate levels, and the presence of ventricular tachycardia (VT) upon ICU admission as independent predictors of mortality in pediatric FM. Additionally, the lactate level at 12 h after initiating ECMO or intensive conventional therapy (ICT) emerged as a reliable indicator of treatment efficacy, closely correlating with mortality risk.

Our study highlights the early recognition of risk factors for death in FM. Initial symptoms are often atypical and non-specific, making timely diagnosis difficult​ [8]. Misdiagnosis rates are high at first presentation, and in some fatal cases, the correct diagnosis was reached only after hemodynamic collapse had already produced seizures, altered consciousness, or dyspnea. Notably, around 70% of children who eventually died from acute myocarditis succumbed within the first week of admission [9]. This rapid trajectory underscores the critical importance of early risk assessment in pediatric FM.

In light of the need for swift intervention, identifying prognostic risk factors at presentation is key to guiding early therapy. We therefore focused on markers obtainable shortly after ICU admission that might stratify mortality risk. Previous studies have linked various biomarkers of organ injury to outcomes in myocarditis, including cardiac enzymes, liver enzymes, renal function, and serum lactate levels [10]. However, in fulminant myocarditis the cascade is typically initiated by extensive myocardial damage, which secondarily causes multi-organ hypoperfusion and injury. It follows that indicators of severe myocardial injury or electrical instability would be the most critical early warning signs of impending clinical deterioration. In line with this, our study found that CK-MB – a sensitive marker of myocardial necrosis – was an independent early predictor of in-hospital mortality in pediatric FM. This finding extends prior observations from smaller studies. For instance, our own earlier work and that of others showed that peak CK-MB measured during ECMO support correlates with mortality, but that pre-ECMO CK-MB was not predictive [11,12]. In contrast, the present multicenter analysis demonstrates that even at ICU admission, an exceedingly high CK-MB can portend poor outcome. We identified a cut-off CK-MB level of 116.8 U/L (nearly five times the upper normal limit) associated with mortality risk. Such an extreme elevation at presentation likely reflects massive myocardial injury and should alert clinicians to a patient at high risk – one who may require escalated support, including consideration for early ECMO, even before further decline occurs.

Life-threatening arrhythmias were another crucial prognostic factor in our cohort. In particular, the occurrence of ventricular tachycardia in the early stage of FM was strongly associated with adverse outcomes. VT signifies severe electrical instability of the myocardium and can rapidly precipitate hemodynamic collapse or sudden cardiac death if not aggressively managed. Clinically, this arrhythmia often serves as a precursor to the need for ECMO support, as it indicates that the myocardium is critically compromised. Our findings are consistent with previous reports linking early arrhythmias to worse outcomes [13]. Similarly, Tuan et al. identified arrhythmia, with VT being the most common presentation, as a key indicator prompting ECMO in children with FM and cardiogenic shock [14]. We also observed in our cohort that cardiac rhythm disturbances could evolve during hospitalization (for example, progressing from complete atrioventricular block to VT), heralding further deterioration; in such scenarios, the initiation of ECMO became imperative to prevent sudden collapse. Importantly, providing ECMO support not only stabilizes circulation but may also facilitate faster recovery of normal rhythm. Prior work has shown that the time to rhythm recovery in FM is shorter with ECMO support compared to conventional therapy​, highlighting an additional benefit of early mechanical support in the context of refractory arrhythmias [15].

Finally, our data reaffirm the central role of serum lactate as a barometer of perfusion and prognostic marker in pediatric FM. Lactate is a well-established, sensitive indicator of tissue hypoperfusion, and numerous studies have confirmed that elevated lactate levels are associated with higher mortality in FM​ [16,17]. Among critically ill children, lactate is one of the most readily available measures of low cardiac output, and its clearance over time reflects the restoration of adequate perfusion [18]. In our cohort, the lactate level at 12 h after starting ECMO or intensive medical therapy proved to be a particularly valuable gauge of treatment efficacy. In this study, elevated lactate levels measured at 12 and 24 h after initiation of treatment were significantly associated with higher mortality, highlighting the prognostic value of persistent hyperlactatemia in pediatric FM. This finding aligns with prior reports in pediatric FM and ECMO populations, where early lactate normalization is associated with better survival [16,17]. For patients managed initially with conventional therapy, failure of lactate to decrease by 12 h should be viewed as a warning sign that current measures are insufficient. In such cases, urgent escalation of support – for example, prompt initiation of ECMO – is warranted rather than waiting until 24 h or later. Conversely, an improving lactate level by 12 h suggests that perfusion is being restored, which correlates with a greater likelihood of survival. Thus, serial lactate monitoring in the first critical hours offers an objective means to evaluate treatment effectiveness and can guide timely decision-making.

The other two parameters (SVO_2_ and urine output) as early treatment response parameters at 12 and 24 h after initiation of ECMO or ICT were also associated with mortality. Although SVO₂ differed significantly between survivors and non-survivors at 12 h, interpretation must consider that values can be influenced by factors such as vasopressor use, hemoglobin concentration, and peripheral shunting. Therefore, SVO₂ should be used in conjunction with other markers of perfusion when evaluating response to therapy. CRRT was used in 24.6% (46/187) of patients, which may have affected urine output measurements. As most patients did not undergo CRRT, urine output was still considered a useful surrogate of systemic perfusion. However, this variable should be interpreted with caution and validated in future stratified analyses.

This study has several limitations. First, its retrospective multicenter design and variation in data collection across institutions resulted in some missing data for key biochemical and hemodynamic indicators. Second, the decision to initiate ECMO or intensive conventional therapy was clinician-dependent, introducing potential selection bias and limiting adjustment for unmeasured confounders. Third, although LVEF was consistently available, other echocardiographic parameters reflecting the severity of FM, such as right ventricular function or pulmonary pressures, were not uniformly recorded. Finally, the predictive model was developed and internally validated within this cohort; external, prospective validation in independent populations is required before clinical implementation.

In summary, this study provides a nuanced understanding of early risk factors in pediatric FM and their clinical implications. By delineating objective indicators available at presentation (such as CK-MB and VT) and early in the course of treatment (12 -h lactate), our findings equip clinicians with practical tools for early risk stratification. Identifying patients at highest risk of deterioration allows for proactive intervention – most importantly, deciding on early ECMO support – which, as our data and prior studies suggest, can dramatically improve survival. These results advance current knowledge by validating specific criteria for early identification of severe FM and emphasizing the crucial window for intervention. Implementing such evidence-based risk assessment in practice could help replicate the improved outcomes observed in our multicenter cohort, ultimately enhancing the management and prognosis of fulminant myocarditis in children.

## CRediT authorship contribution statement

QS, RL, LY, ZL and DM designed the study. LY and ZL performed the statistical analysis and wrote the manuscript. WZ, XM, YZ, JW, YC, YG, WW and ZL contributed to the data analysis, visualization and interpretation. All authors contributed to the critical review and provided final approval of the manuscript. The decision to submit the manuscript was made collectively by all authors.

## Consent for publication

Not applicable.

## Ethical approval

This study was approved by the Ethics Committee of the Children’s Hospital of Zhejiang University, School of Medicine, Zhejiang, China(2020-IRB-006) and the requirement for informed patient consent was waived in view of the retrospective nature of the study.

## Funding

This study is funded by the innovation design projects of the National Clinical Research Center for Child Health (G20B0006), the Central Guiding Fund for Local Science and Technology Development Projects (2023ZY1058), the National Natural Science Foundation of China (82470544), the Fundamental Research Funds for the Central Universities (226-2024-00153), and the Open Project Fund of Henan Provincial Research Centerfor Precision Diagnosis and Treatment of Pulmonary Diseases Based on Multi-omics (DZXGCZXKF04).

## Availability of data and materials

The datasets used and analyzed in this study are available from the corresponding author upon reasonable request.

## Declaration of competing interest

The authors declare that they have no competing interests.
